# A Red Emissive Fluorescent Turn-on Sensor for the Rapid Detection of Selenocysteine and Its Application in Living Cells Imaging

**DOI:** 10.3390/s20174768

**Published:** 2020-08-24

**Authors:** Zongcheng Wang, Huihuang Zheng, Chengliang Zhang, Dongfang Tang, Qiyao Wu, Wubliker Dessie, Yuren Jiang

**Affiliations:** 1 College of Chemistry and Chemical Engineering, Central South University, Changsha 410083, China; wangzongch@huse.edu.cn (Z.W.); zcl525@csu.edu.cn (C.Z.); yaoyaowu@csu.edu.cn (Q.W.); 2 College of Chemical and Biological Engineering, Hunan University of Science and Engineering, Yongzhou 425199, China; zaun1999@163.com (H.Z.); tangdf@smail.hunnu.edu.cn (D.T.); wubliker@njtech.edu.cn (W.D.)

**Keywords:** fluorescent sensor, selenocysteine, long-wavelength, living cell fluorescence imaging

## Abstract

The content of selenocysteine in cells has an important effect on a variety of human diseases, and the detection of selenocysteine by fluorescent sensors in vivo has shown many advantages. In order to further develop fast-reaction-time, good-selectivity, and high-sensitivity long-wavelength selenocysteine fluorescent sensors, we designed and synthesized the compound **YZ-A4** as a turn-on fluorescent sensor to detect the content of selenocysteine. The quantitative detection range of the sensor **YZ-A4** to selenocysteine was from 0 to 32 μM, and the detection limit was as low as 11.2 nM. The sensor displayed a rapid turn-on response, good selectivity, and high sensitivity to selenocysteine. Finally, we have demonstrated that **YZ-A4** could be used for fluorescence imaging of selenocysteine in living cells.

## 1. Introduction

Although there are very few selenium (Se) requirements, it is essential for human health and development [1]. Insufficient or excessive intake of selenium can lead to many diseases [2,3,4]. Selenocysteine (Sec) is the main form of selenium compounds in the cell [5]. Known as the 21st amino acid in protein synthesis, Sec is an important component of selenium protein (SeP), which has high enzyme catalytic activity [6]. The SeP enzyme is involved in the body’s antioxidant defense, growth, muscle development, thyroid hormone regulation, and immune function, as well as other physiological functions of metabolism [7,8]. As the reactive site of SeP, Sec content is closely related to the high catalytic capacity of SeP and many physiological functions such as cancer, cardiovascular disease, diabetes, neurodegenerative disease, and male infertility [9,10,11]. Due to the important role of Sec in human physiological function, it is necessary to develop a fast and reliable method to detect Sec in biological systems [12].

Compared to high-performance liquid chromatography (HPLC), gas chromatography (GC), and inductively coupled plasma mass spectrometry (ICP-MS) detection methods, fluorescence sensors can avoid the efficient extraction of Sec and the destruction of the biological sample [13,14]. They are suitable for the detection of Sec in a biological system with high sensitivity and selectivity, and fast response [15]. Therefore, the detection of Sec in biological samples by the fluorescent sensor method has attracted the extensive attention of researchers [16].

Recently, several Sec fluorescent sensors including nanosensors were reported, using 2,4-dinitrophenyl ether, 2,4-dinitrobenzene sulfone amide, or 2,4-dinitrobenzene sulfonate ester as the reaction sites [17,18,19,20]. Among them, 2,4-dinitrobenzene sulfonate ester has a faster response time [19]. At the same time, based on luciferin, coumarin, hemicyanine, and xanthene dyes, many fluorescent sensors were developed to detect Sec [17,21,22,23,24]. In the design process, fluorescent sensors with long-wavelength emission are preferred to reduce phototoxicity and increase tissue penetration [12,18]. 

**Scheme 1:**
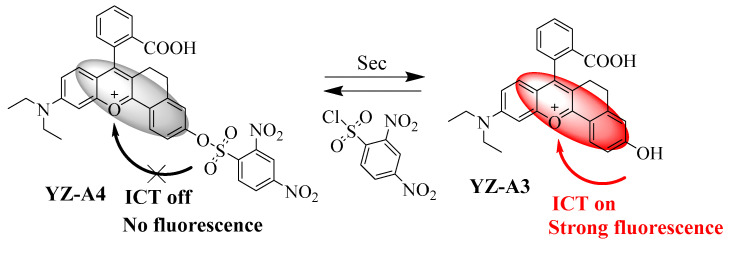
The detection process of **YZ-A4** toward selenocysteine (Sec).

Though a few long-wavelength Sec fluorescent sensors have been reported in recent years [24,25,26], further development research is still needed to date, such as developing fast-reaction-time, good-selectivity, and high-sensitivity long-wavelength Sec fluorescent sensors. Hence, we rationally designed a novel turn-on sensor **YZ-A4** based on xanthene dyes, and using a 2,4-dinitrobenzene sulfonate ester as the reaction sites of Sec. The sensor exhibited excellent optical properties, fast reaction time, good selectivity, and high sensitivity to Sec. Finally, the sensor **YZ-A4** was successfully applied to detect Sec for fluorescence imaging in biological samples (Scheme 1).

## 2. Materials and Methods

### 2.1. Chemicals and Apparatus

All chemicals were acquired from Shanghai Titan Technology Co., LTD and used without further purification. ^1^H NMR (400 MHz) and ^13^C NMR (100 MHz) spectra were performed on a Bruker Advance III HD400 spectrometer. The HRMS spectra were recorded with an Agilent 6540. The HPLC spectra were measured with a SHIMADZU SPD-10ATVP. UV-visible absorption and fluorescence spectra were recorded with a SHIMADZU UV-2600 and SHIMADZU RF-6000 at room temperature, respectively.

### 2.2. Synthesis of Dye YZ-A3 and the Fluorescent Sensor YZ-A4

According to the literature method, the dye **YZ-A3** was synthesized [27,28]. 2-(4-(Diethylamino)-2-hydroxybenzoyl)benzoic acid (313 mg, 1 mmol) and 6-hydroxy-3,4-dihydronaphthalen-1(2H)-one (162 mg, 1 mmol) were added to the stirred solution of concentrated H_2_SO_4_ (15 mL) in a flask. The mixture was heated at 90 °C for 2.5 h, cooled to room temperature, and poured into ice (100 g). After 70% HClO_4_ (1 mL) was added to the solution, the red precipitate gradually generated, which was filtered off and washed with ice water (4 × 5 mL). The precipitate was purified through the a silica gel chromatography column with v(CH_2_Cl_2_)/v(CH_3_OH) (18:1) as the eluent, and **YZ-A3** was obtained as a brown solid (388 mg, 72%). ^1^H NMR (400 MHz, DMSO-*d*_6_) δ 13.31 (s, 1H), 11.40 (s, 1H), 8.27 (s, 1H), 8.11–8.13 (m, 1H), 7.83 (t, *J* = 7.5 Hz, 1H), 7.73 (t, *J* = 7.6 Hz, 1H), 7.38 (*d*, *J* = 7.4 Hz, 1H), 7.26–6.95 (m, 2H), 6.79–6.84 (m, 2H), 3.52–3.63 (m, 4H), 2.74–2.83 (m, 2H), 2.21–2.31 (m, 2H), 1.22–1.11 (m, 6H). ^13^C NMR (100 MHz, DMSO-*d*_6_) δ 198.75, 167.44, 166.94, 165.10, 164.65, 157.64, 154.74, 153.96, 145.50, 140.43, 134.70, 133.58, 132.51, 131.40, 130.33, 130.11, 128.12, 118.03, 116.20, 109.66, 104.47, 96.71, 45.64, 44.49, 26.69, 23.59, 12.90. HRMS (ESI): m/z calculated for (C_28_H_26_NO_4_)^+^: 440.1856, found: 440.1854.

The fluorescent sensor **YZ-A4** was synthesized according to the literature [29]. Compound **YZ-A3** (0.162 g, 0.3 mmol) and 2,4-dinitrobenzene sulfonyl chloride (0.080 g, 0.3 mmol) were stirred in dry dichloromethane (10 mL) at room temperature under N_2_ for 4 h. After the solution was condensed and purified by a silica gel chromatography column with CH_2_Cl_2_/EtOH (20:1) as the eluent, a purple solid (0.157 g, 68%) was obtained. ^1^H NMR (400 MHz, DMSO-*d*_6_) δ 9.13 (d, *J* = 2 Hz, 1H), 8.61 (dd, *J* = 9, 2 Hz, 1H), 8.28 (d, *J* = 9 Hz, 1H), 7.96 (d, *J* = 8 Hz, 1H), 7.89 (d, *J* = 9 Hz, 1H), 7.78 (t, *J* = 7 Hz, 1H), 7.68 (t, *J* = 7 Hz, 1H), 7.34 (d, *J* = 8 Hz, 1H), 7.19–7.09 (m, 2H), 6.59–6.41 (m, 3H), 3.31–3.36 (m, 4H), 2.75 (dd, *J* = 15, 8 Hz, 2H), 2.03–1.92 (m, 1H), 1.80–1.67 (m, 1H), 1.09 (t, *J* = 7 Hz, 6H). ^13^C NMR (100 MHz, DMSO-*d*_6_) δ 169.42, 151.99, 149.12, 148.57, 139.80, 135.85, 134.12, 131.15, 130.51, 128.90 127.95, 127.06, 124.95, 124.76, 124.59, 124.17, 121.64, 120.48, 109.84, 97.31, 44.23, 27.04, 20.78, 12.78. HRMS (ESI): m/z calculated for (C_34_H_28_N_3_O_10_S)^+^: 670.1490, found: 670.1485.

### 2.3. Imaging Application of the Fluorescent Sensor YZ-A4 in A549 Cells

A549 cells (human lung cancer cells) were incubated in 35 mm cell culture dishes in the 1640 culture medium with 10% fetal bovine serum, 1% penicillin, and 1% streptomycin, under the atmosphere of 5% CO_2_ at 37 °C in a carbon dioxide incubator for 24 h. L-Selenocystine (Sec)_2_ (final concentration: 10 μM) was added as a source of Sec from a 1 mM stock in PBS (10 mM, pH 7.40). After sensor **YZ-A4** (final concentration: 10 μM, containing 1% DMSO) was added from a 1 mM stock in DMSO, cells were incubated for different times and subsequently washed with PBS (pH = 7.4) three times. Imaging application of the fluorescent sensor **YZ-A4** was completed with a Nikon Ni-U fluorescence microscope (red channel, excitation from 540 to 565 nm and emission from 605 to 655 nm).

## 3. Results

### 3.1. Design and Synthesis of the Fluorescent Sensor YZ-A4

Based on the previous studies [24], we modified the fluorophore structure by attaching a benzene ring to the xanthene fluorophore to stabilize the positive ions. Then, the 2,4-dinitrobenzene sulfonate ester group with a fast reaction rate was selected to detect Sec as the reaction sites. Due to the 2,4-dinitrobenzene sulfonate ester group truncating the intramolecular charge transfer (ICT) effect [22,30], the fluorescence of **YZ-A4** quenched. When reacted with Sec, the ICT effect of the generated **YZ-A3** was restored. Therefore, **YZ-A4** can be theoretically used to detect Sec rapidly by the "turn-on" fluorescence reaction under simulated physiological conditions.

**Scheme 2:**
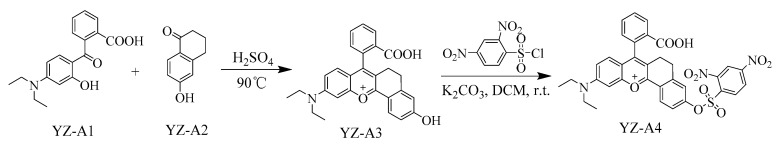
Synthesis of YZ-A4.

A fluorescent sensor **YZ-A4** was synthesized as shown in Scheme 2. First, **YZ-A3** dye was synthesized by a cyclization reaction of **YZ-A1** with **YZ-A2** in concentrated H_2_SO_4_ at 90 °C. Next, the **YZ-A3** dye reacted with 2,4-dinitrobenzenesulfonyl chloride under room temperature conditions to give the fluorescent sensor **YZ-A4**. The structure of **YZ-A3** dye and fluorescent sensor **YZ-A4** was characterized by ^1^H NMR, ^13^C NMR, and HRMS spectra in Figures S1–S6 in the Supplementary Materials.

### 3.2. Optical Properties of Dye YZ-A3 and the Fluorescent Sensor YZ-A4

**Figure 1:**
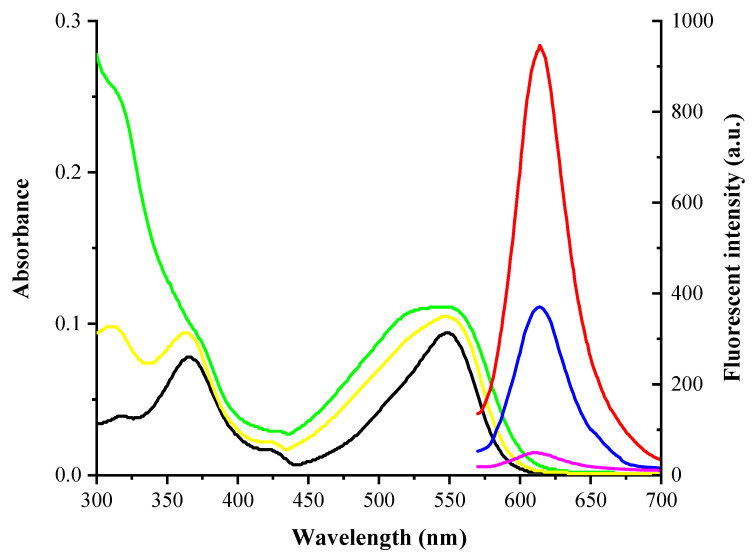
Absorption spectra of 10 μM **YZ-A3** (black) and **YZ-A4** (green), and treating **YZ-A4** with 10 μM Sec (yellow) in PBS (10 mM, pH 7.40, containing 1% DMSO as cosolvent). Fluorescence spectra of 10 μM **YZ-A3** (red), and **YZ-A4** before (pink) and after (blue) reaction with 10 μM Sec in PBS (10 mM, pH 7.40, containing 1% DMSO as cosolvent), excited at 550 nm.

To determine the optical properties of the fluorescent sensor **YZ-A4**, the UV-visible absorption and fluorescence spectrum of the **YZ-A3** dye (10 μM) and sensor **YZ-A4** (10 μM) were examined under the PBS buffer (10 mM, pH 7.4, containing 1% DMSO). The **YZ-A3** dye and sensor **YZ-A4** both display a maximum absorption peak at around 550 nm, but the **YZ-A3** dye displays a maximum absorption peak at 365 nm (black), which can be used to distinguish them in Figure 1. At the same time, after treating **YZ-A4** with Sec (10 μM), a new maximum absorption peak appeared at 365 nm, too (yellow). When excited at 550 nm, the **YZ-A3** dye displays strong fluorescence at 614 nm (red), while **YZ-A4** displays almost no fluorescence (pink). However, after treating **YZ-A4** with Sec (10 μM), a new fluorescence peak arose at 614 nm (blue). The spectrogram indicated that **YZ-A4** had reacted with Sec, which resulted in the fracture of the sulfonate ester group and the formation of the **YZ-A3** fluorophore, recovering the ICT effect of **YZ-A3** and producing strong fluorescence. HPLC analysis showed that after treating **YZ-A4** with Sec (10 μM), a new chromatographic peak was generated with the same retention time as **YZ-A3** (5.106 min, blue) in Figure S7 in the Supplementary Materials, which proved that the reaction mechanism can induce the production of **YZ-A3**.

**Figure 2:**
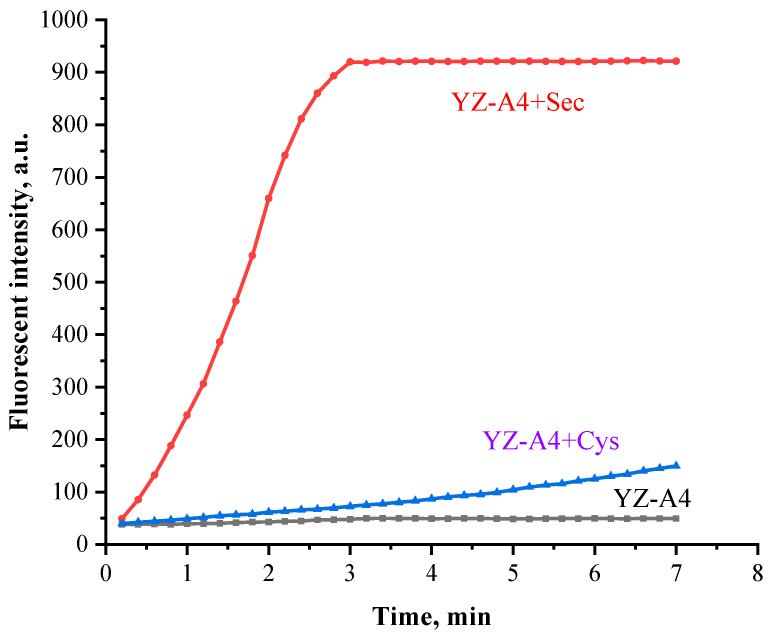
Time-dependent fluorescent intensity of 10 µM **YZ-A4** at 614 nm in the absence (black) and presence of 100 μM Sec (red), and presence of 100 μM Cys (blue) in PBS (10 mM, pH 7.40, containing 1% DMSO as cosolvent), excited at 550 nm.

In order to determine the optimal reaction time, we studied the effect of reaction time on the fluorescence intensity of **YZ-A4** reacted with Sec. Due to the instability, Sec was freshly prepared by mixing L-selenocysteine with dithiothreitol each time [29]. The response of **YZ-A4** (10 μM) to fresh Sec (100 μM) was very rapid and completed within about 3 min and the fluorescence enhancement was 20-fold (Figure 2). As cysteine (Cys) and Sec are very similar in structure, the reactivity of **YZ-A4** with Cys (100 μM) within 7 min was determined. Fluorescence enhancement was not obvious after **YZ-A4** reacted with Cys within 3 min.

**Figure 3:**
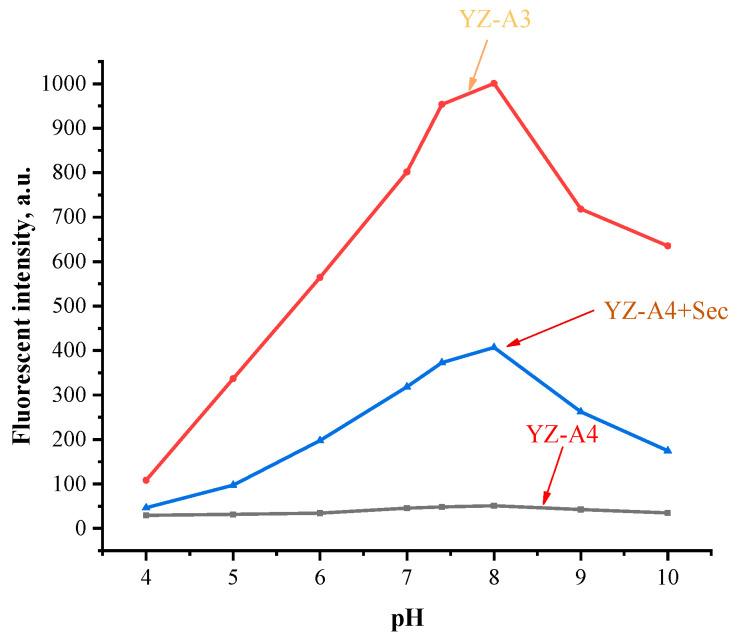
Fluorescent intensity of 10 μM **YZ-A3** (red), and **YZ-A4** before (black) and after (blue) reaction with 10 μM Sec in different PBS (pH 4–10, 10 mM, containing 1% DMSO as cosolvent). Excitation at 550 nm and emission at 614 nm.

To further determine the appropriate reaction pH value of **YZ-A4** and Sec, we studied the fluorescence intensity changes of the **YZ-A4** sensor, treating **YZ-A4** with Sec and the **YZ-A3** dye under different pH conditions. As shown in Figure 3, under different pH conditions, there is almost no fluorescence with **YZ-A4**. However, the fluorescence intensity enhanced after **YZ-A4** reacted with Sec. Moreover, with the increase in pH value from 4.0 to 8.0, the fluorescence intensity of **YZ-A4** reacted with Sec also increased, but slightly decreased after a pH value more than 8.0. These changes are consistent with the **YZ-A3** dye, which suggested that **YZ-A4** reacted with Sec had a turn-on response.

**Figure 4:**
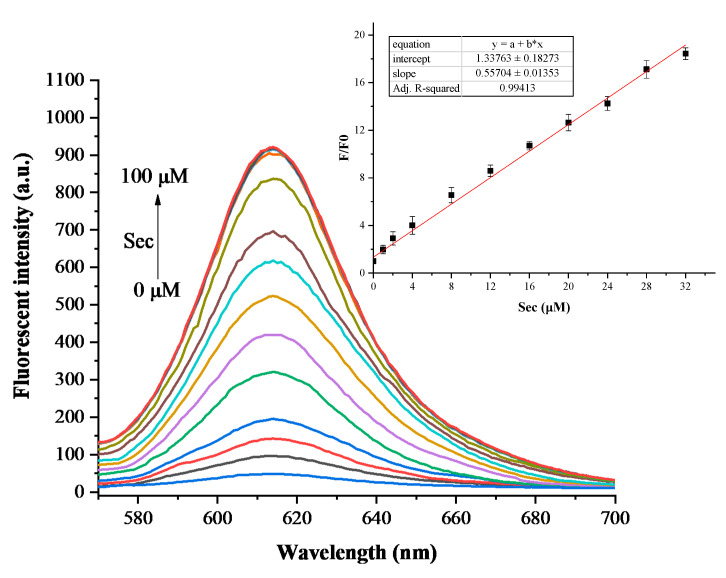
Fluorescence responses of 10 µM **YZ-A4** to different Sec concentrations (0, 1, 2, 4, 8, 12, 16, 20, 24, 28, 32, 40, 50, 75, 100 µM) in PBS (10 mM, pH 7.40, containing 1% DMSO as cosolvent). The inset displays the linear relationship of the fluorescent intensity at 614 nm, excited at 550 nm.

The detection range of **YZ-A4** to Sec concentration was studied. With the increase in Sec concentrations (0–100 μM), the fluorescence intensity of **YZ-A4** reacted with Sec considerably increased, as shown in Figure 4. A good linearity response was observed for Sec in the concentration range 0–32 µM, and the regression equation was F/F_0_ = 0.55704 [Sec] μM + 1.33763 with R^2^ = 0.99413. Based on the regression equation of the response of **YZ-A4** to Sec and standard deviation of blank parallel determination, the limit of detection was calculated as 11.2 nM.

### 3.3. The Anti-Interference Ability and Selectivity of YZ-A4 toward Sec

**Figure 5:**
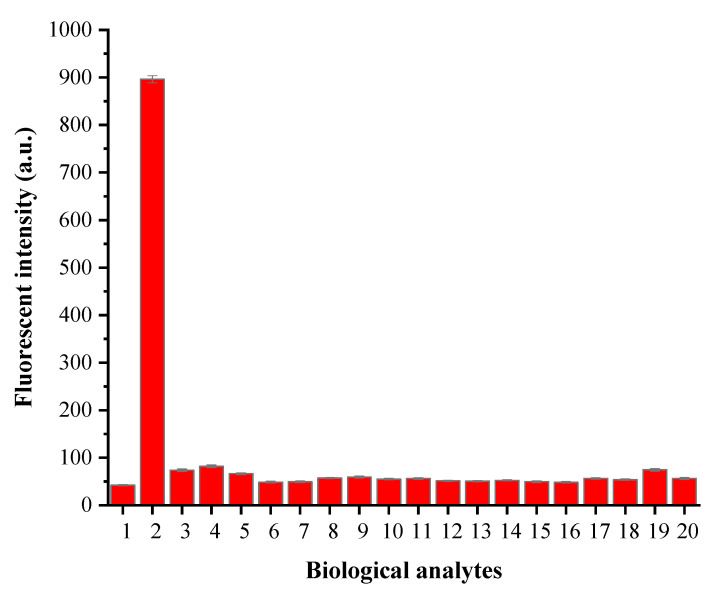
Fluorescent intensity responses of 10 μM **YZ-A4** at 614 nm to biological analytes (100 μM) in PBS (10 mM, pH 7.40, containing 1% DMSO as cosolvent). Legend: (1) Blank; (2) Sec; (3) Cys; (4) Hcy; (5) GSH; (6) Glu; (7) Asp; (8) Val; (9) Phe; (10) Pro; (11) Thr; (12) Arg; (13) Lso; (14) Leu; (15) His; (16) Lys; (17) Try; (18) Ser; (19) Na_2_Se; (20) Na_2_SeO_3_. Excitation at 550 nm. Each piece of data was obtained 3 min after mixing.

The selectivity of **YZ-A4** toward Sec was evaluated in the physiological pH value environment. Due to the difference in reaction rate, biothiols (Cys, Hcy and GSH) could not distinctly enhance the fluorescence intensity of **YZ-A4** within 3 min, showing a certain selectivity, which is consistent with the reported sensor by Zhang et al. [29]. At the same time, the sensor shows better selectivity to Sec than many amino acids (Glu, Asp, Val, Phe, Pro, Thr, Arg, Lso, Leu, His, Lys, Try, and Ser) and other selenocompounds (Na_2_Se and Na_2_SeO_3_) in Figure 5. The results indicated that the sensor **YZ-A4** could particularly recognize Sec under physiological pH value conditions. In addition, the anti-interference ability of **YZ-A4** to the biological analytes was evaluated. In the presence of other bioactive substances, the fluorescence intensity of the **YZ-A4** sensor to Sec changed little, which demonstrated that other bioactive substances had no interference when the **YZ-A4** sensor detected Sec, as shown in Figure S8 in the Supplementary Materials.

### 3.4. Cell Imaging

**Figure 6:**
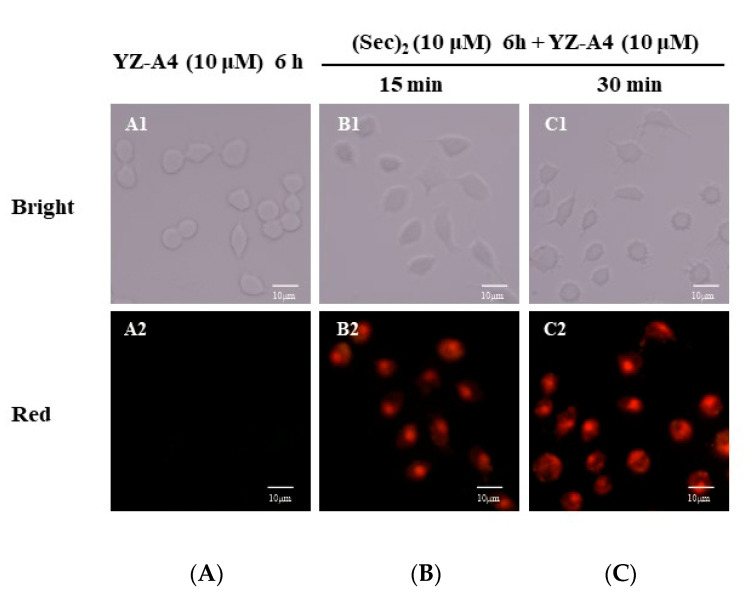
Fluorescence images of living A549 cells. The fluorescence was measured at a red channel. (**A**) 10 μM **YZ-A4** for 6 h, (**B**) 10 μM (Sec)_2_ for 6 h + 10 μM **YZ-A4** for 15 min, (**C**) 10 μM (Sec)_2_ for 6 h + 10 μM **YZ-A4** for 30 min. The scale bar is 10 μm. Red channel, excitation from 540 to 565 nm and emission from 605 to 655 nm.

We selected A549 cells as a test model to test the recognition capability of **YZ-A4** toward Sec in living cells. The Sec inside cells can be produced by the reaction of (Sec)_2_ with intracellular biothiols [29]. As shown in Figure 6, A549 cells incubated with the sensor **YZ-A4** (10 μM) showed hardly any fluorescence emission after 6 h, which suggested that **YZ-A4** was not responsive to the biothiols in cells. (Figure 6, panel A2). However, when cells were incubated with (Sec)_2_ (10 μM) for 6 h and the sensor **YZ-A4** (10 μM) was then added for 15 min, red fluorescence appeared (Figure 6, panel B2). After the sensor **YZ-A4** was added for 30 min, strong fluorescence was significantly produced and no longer enhanced (Figure 6, panel C2). These results suggest that the biothiols species did not exhibit interference to the detection of Sec, and **YZ-A4** is a suitable tool for detecting Sec in living cells. At the same time, the concentration of Sec in normal cells is very low. However, when there are some physiological abnormalities, such as thyroid disease, the concentration of Sec will increase significantly, and the fluorescence sensors can detect the Sec concentration [23,26]. Therefore, **YZ-A4** can be used to detect Sec concentration in abnormal cells of simulated physiological diseases.

## 4. Conclusions

In summary, a novel red emissive fluorescent sensor **YZ-A4** was designed and synthesized for the fast, accurate, and highly selective detection of Sec in living cells. The response of **YZ-A4** to Sec was completed to produce red fluorescence within about 3 min, and the fluorescence enhancement at 614 nm was 20 fold under simulated physiological conditions. The quantitative detection range of the sensor **YZ-A4** to Sec was from 0 to 32 μM, and the detection limit was as low as 11.2 nM, whose detection limit was lower and the response time was faster than those of the reported sensors [12,31]. The sensor **YZ-A4** exhibited good selectivity to Sec, whereas the biothiols and other amino acids hardly exhibited interference. The sensor **YZ-A4** could be used for fluorescence imaging of Sec in living A549 cells. The performance of the fluorescent sensor indicated that the sensor can be used as a fast and accurate detection tool in detecting Sec in living cells samples.

## Supplementary Materials

The following are available online at https://www.mdpi.com/1424-8220/20/17/4768/s1, Figure S1: ^1^H NMR spectrum of compound **YZ-A3** (DMSO-*d*_6_), Figure S2: ^13^C NMR spectrum of compound **YZ-A3** (DMSO-*d*_6_), Figure S3: HRMS spectrum of compound **YZ-A3**, Figure S4: ^1^H NMR spectrum of compound **YZ-A4** (DMSO-*d*_6_), Figure S5: ^13^C NMR spectrum of compound **YZ-A4** (DMSO-*d*_6_), Figure S6: HRMS spectrum of compound **YZ-A4**, Figure S7: HPLC spectrum of compound **YZ-A3** (black) and **YZ-A4** (pink), and treating **YZ-A4** with Sec (blue), Figure S8: Fluorescent intensity responses of 10 μM **YZ-A4** at 614 nm to Sec (100 μM) in the presence of various biological analytes (100 μM) in PBS (10 mM, pH 7.40, containing 1% DMSO as cosolvent). Legend: (1) Blank; (2) Cys; (3) Hcy; (4) GSH; (5) Glu; (6) Asp; (7) Val; (8) Phe; (9) Pro; (10) Thr; (11) Arg; (12) Lso; (13) Leu; (14) His; (15) Lys; (16) Try; (17) Ser; (18) Na_2_Se; (19) Na_2_SeO_3_. Excitation at 550 nm. Each piece of data was obtained 3 min after mixing. Table S1: The performance parameters of some reported Sec fluorescent sensors.
