# Supplementary material for: A Red Emissive Fluorescent Turn-on Sensor for the Rapid Detection of Selenocysteine and Its Application in Living Cells Imaging
Source: Sensors (Basel). 2020 Aug 24;20(17):4768. doi: 10.3390/s20174768 (PMC7506812; doi:10.3390/s20174768)
Supplement: Supplementary file 1 [file sensors-20-04768-s001.pdf]

A Red Emissive Fluorescent Turn-on sensor for the Rapid Detection of Selenocysteine and Its Application in Living Cells Imaging

Supplementary Materials

Zongcheng Wang[1,2], Huihuang Zheng[2], Chengliang Zhang[1], Dongfang Tang[2], Qiyao Wu[1], Wubliker Dessie[2], Yuren Jiang[1,*]

[1] College of Chemistry and Chemical Engineering, Central South University, Changsha, 410083, China

[2] College of Chemical and Biological Engineering, Hunan University of Science and Engineering, Yongzhou, 425199, China

Correspondence to:

*Yuren Jiang

College of Chemistry and Chemical Engineering, Central South University, Changsha 410083, Hunan, China

Telephone number: 86-0731-86781389

Fax number: 86-0731-88859988

E-mail: jiangyr@mail.csu.edu.cn

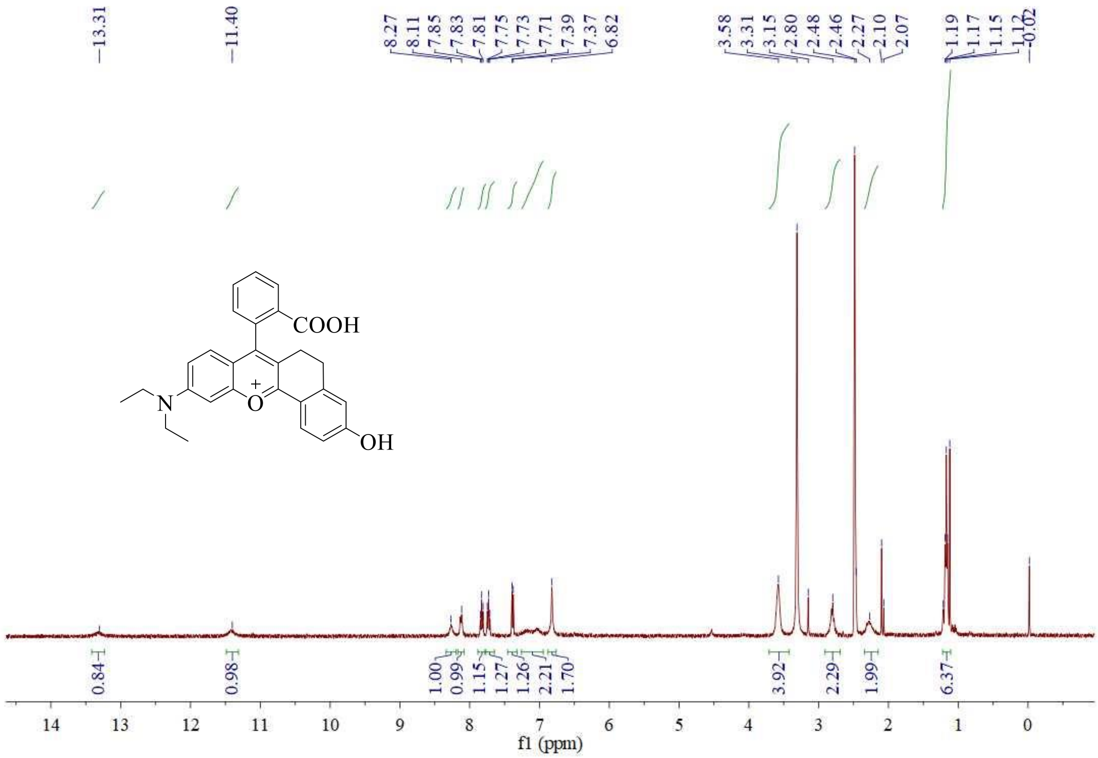

Figure S1. ^{1}H NMR spectrum of compound **YZ-A3** (DMSO-d_6)

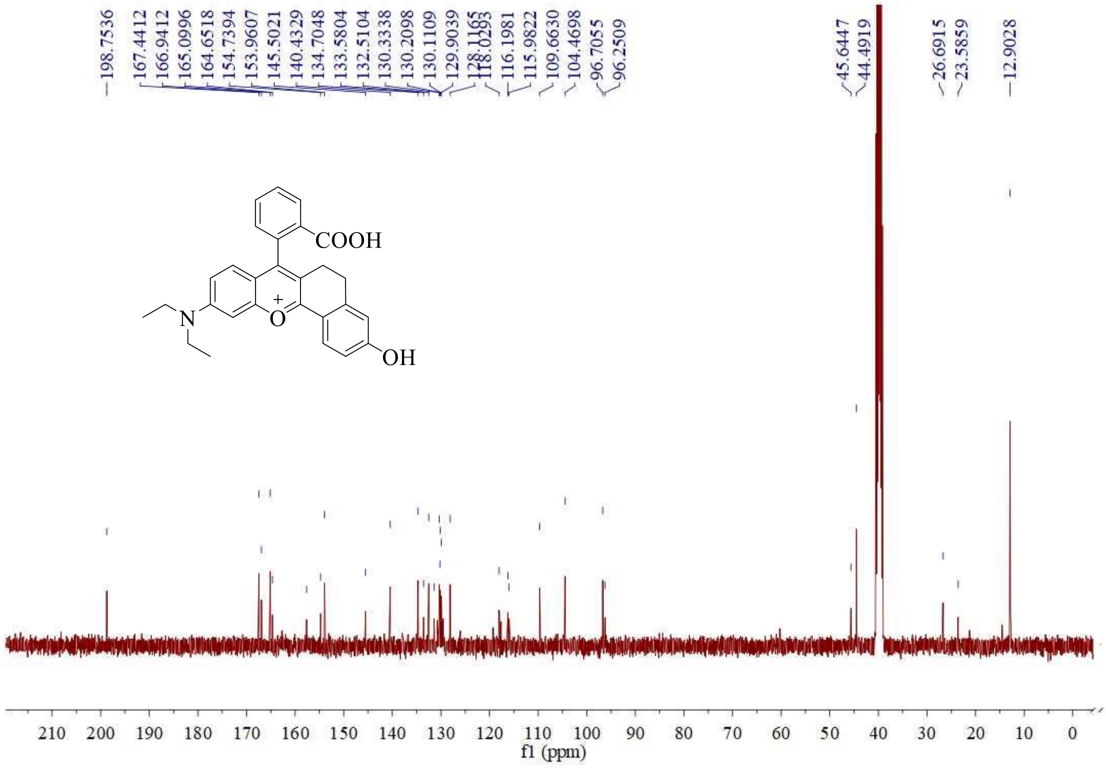

Figure S2. ^{13}C NMR spectrum of compound **YZ-A3** (DMSO-d_6)

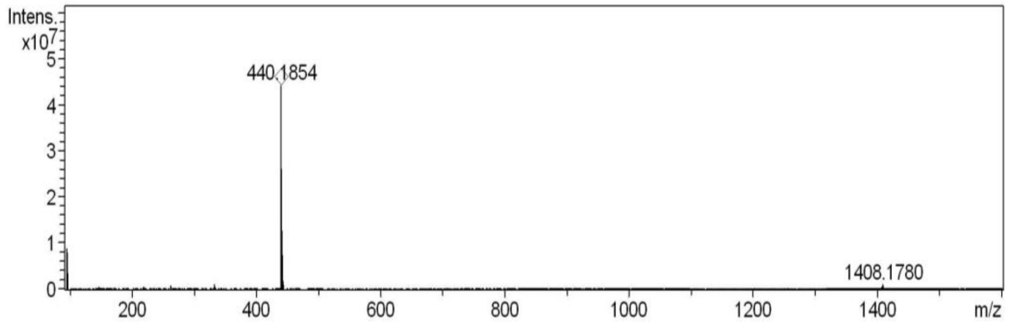

Figure S3. HRMS spectrum of compound **YZ-A3**

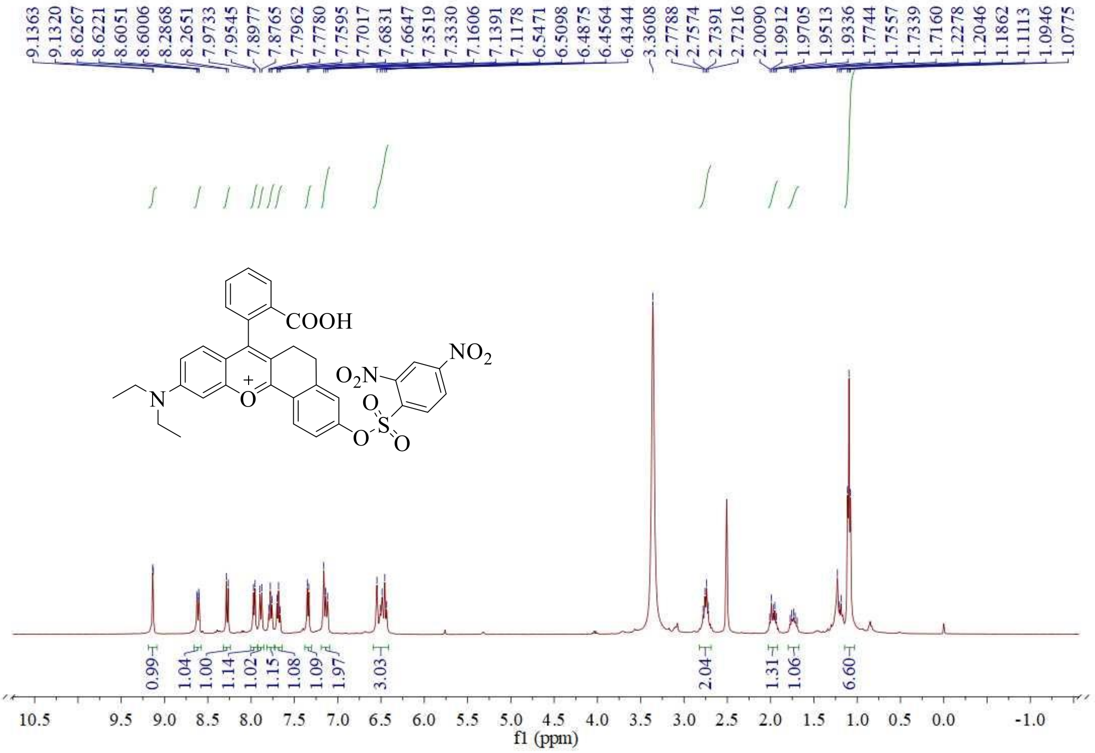

Figure S4. ^{1}H NMR spectrum of compound **YZ-A4** (DMSO-d_6)

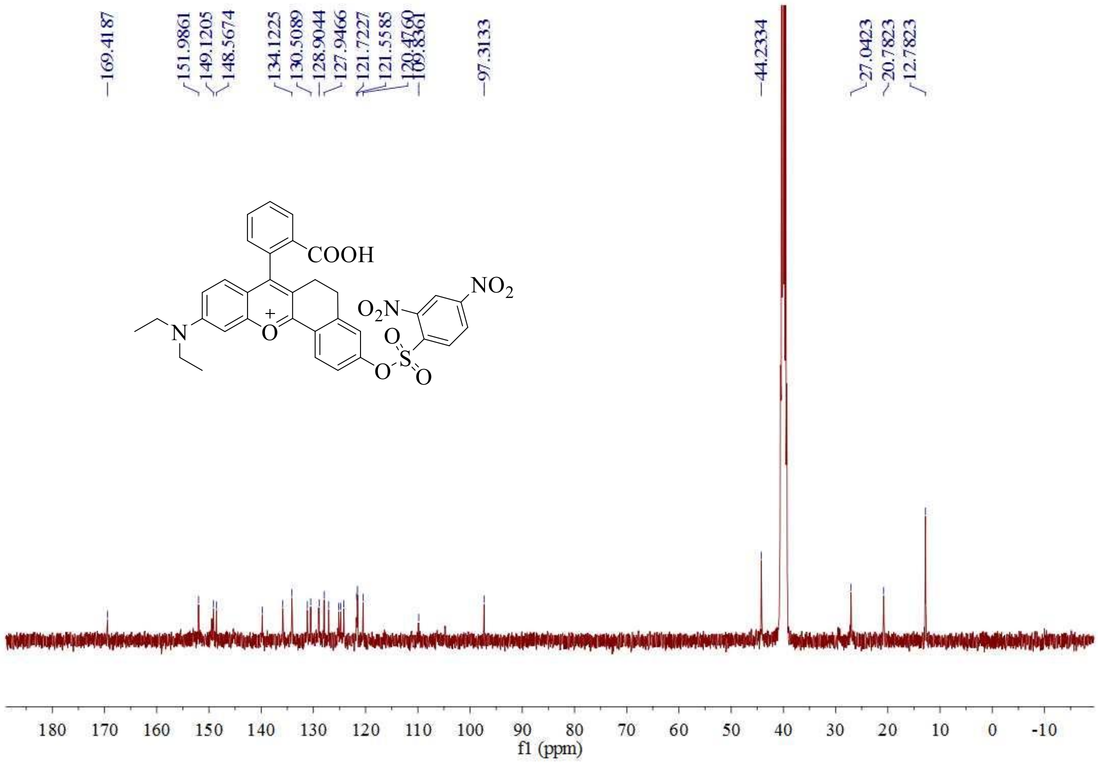

Figure S5. ^{13}C NMR spectrum of compound **YZ-A4** (DMSO-d_6)

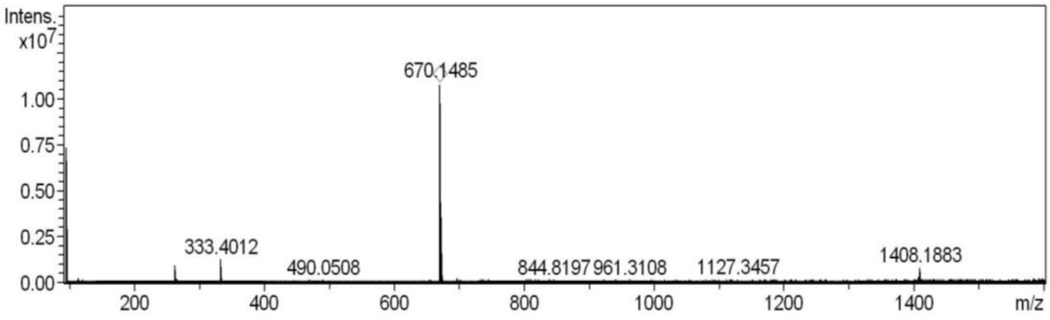

Figure S6. HRMS spectrum of compound **YZ-A4**

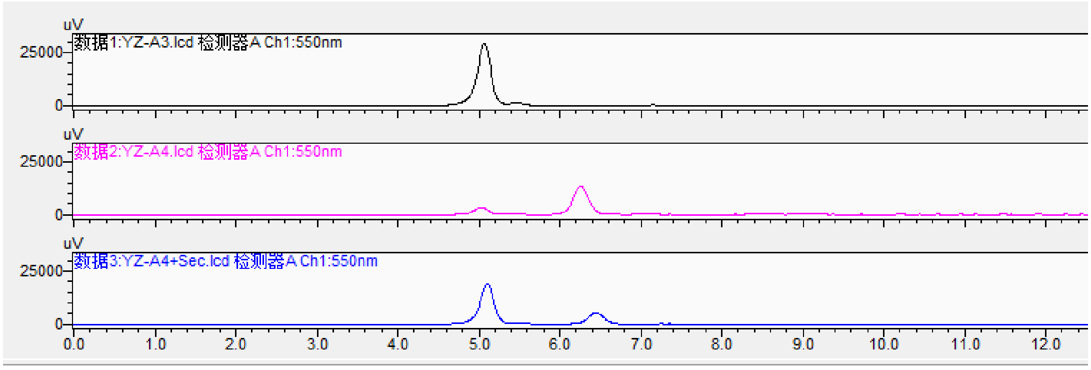

Figure S7. HPLC spectrum of compound **YZ-A3** (black)**, YZ-A4** (pink) and treating **YZ-A4** with Sec (blue).

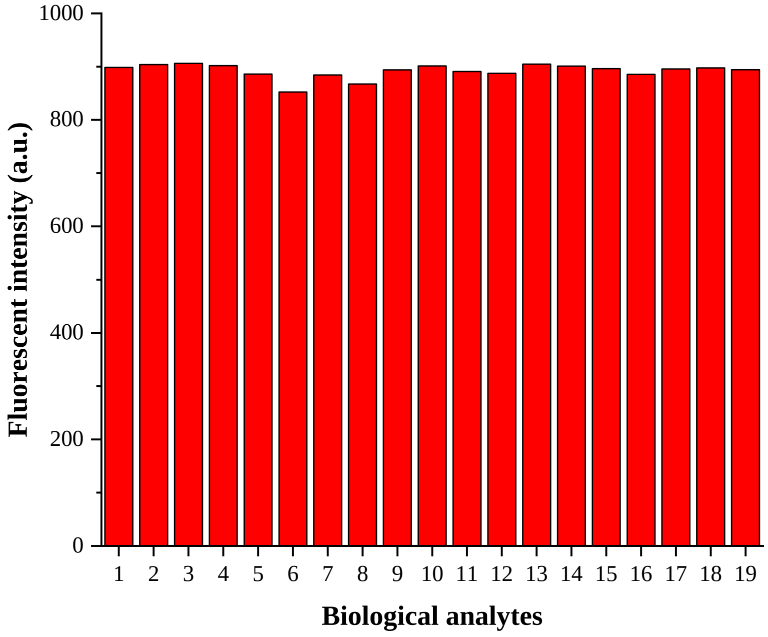

Figure S8. Fluorescent intensity responses of 10 μM **YZ-A4** at 614 nm to Sec (100 μM) in the presence of various biological analytes (100 μM) in PBS (10 mM, pH 7.40, containing 1% DMSO as cosolvent). Legend: (1) Blank; (2) Cys; (3) Hcy; (4) GSH; (5) Glu; (6) Asp; (7) Val; (8) Phe; (9) Pro; (10) Thr; (11) Arg; (12) Lso; (13) Leu; (14) His; (15) Lys; (16) Try; (17) Ser; (18) Na_2Se; (19) Na_2SeO_3. Excitation at 550 nm. Each data was obtained 3 min after mixing.

Table S1. The performance parameters of some reported Sec fluorescent sensors.

Sensors	Structure	λ_{Ex}/nm	λ_{Em}/nm	λ_{Stokes}/nm	LOD/nM	Responsetime/min	Bioimaging	Ref
This work	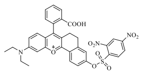	550	614	64	11.2	3	A549	-
GQ-Sec	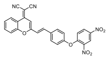	560	706	146	62	5	HeLa	[12]
HD-Sec	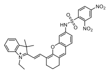	650	712	62	-	10	MCF-7/Mice	[18]
Sel-green	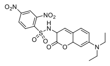	370	502	132	62	3	HepG2	[21]
HB	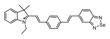	460	580	120	7	10	HepG2	[23]
O-hNRSel	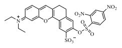	540	624	84	17.4	2	HeLa	[24]
Sel-p1	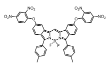	594	663	69	16	15	MCF-7/Mice	[25]
Sel-p2	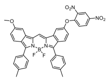	594	655	61	9	15	MCF-7/Mice	[25]
GF-Sec	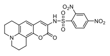	380	535	155	18	8	A549/Zebrafish	[31]
